# Insulin control in fruit flies

**DOI:** 10.7554/eLife.106220

**Published:** 2025-03-13

**Authors:** Omowumi Kayode

**Affiliations:** 1 Department of Biochemistry, Mountain Top University Makogi Oba Nigeria

**Keywords:** metabolism, insulin-producing cells, biogenic amines, neuropeptides, neuroendocrine regulation, *D. melanogaster*

## Abstract

Investigating how the production of insulin is regulated in fruit flies reveals surprising insights that may help to better understand how this process unfolds in humans.

**Related research article** Held M, Bisen RS, Zandawala M, Chockley AS, Balles IS, Hilpert S, Liessem S, Cascino-Milani F, Ache JM. 2025. Aminergic and peptidergic modulation of insulin-producing cells in *Drosophila*. *eLife* **13**:RP99548. doi: 10.7554/eLife.99548.

In humans, the pancreas plays a crucial role in regulating blood sugar levels by producing a hormone called insulin. In fruit flies, cells in the brain called insulin-producing cells (IPCs for short) perform a similar function. These tiny insects may seem like an unlikely choice for studying insulin regulation, but their biology shares many similarities with humans. By studying fruit flies, scientists can gain valuable insights into complex biological processes, including metabolism, insulin production and diseases like diabetes (Marder, 2012; Taghert and Nitabach, 2012).

Whether in insects or mammals, maintaining stable blood sugar levels requires insulin release to be carefully regulated (Meyer et al., 2021). In fruit flies, chemicals called neuromodulators ‘tell’ IPCs when and how to produce the hormone. This involves small protein-like molecules (known as neuropeptides), as well as biogenic amines such as dopamine and serotonin, binding to receptors on the surface of the cells. Each type of neuromodulator can increase or decrease insulin release, depending on what the body needs (Taghert and Nitabach, 2012). Yet, precisely how neuromodulators interact with IPCs in fruit flies is still poorly understood; in particular, it remains unclear whether these cells exhibit functional diversity – that is, whether individual IPCs respond to various neuromodulatory signals in different ways. Now, in eLife, Jan Ache and colleagues – including first authors Martina Held, Rituja Bisen and Meet Zandawala – report a comprehensive analysis of how IPCs are controlled in fruit flies (Held et al., 2025).

The team, who are based at Julius-Maximilians-University of Würzburg, the University of Nevada Reno and Charité–Universitätmedizin Berlin, used a variety of techniques to manipulate and monitor brain signals in the insects. This allowed them to observe how both individual cells and populations of IPCs responded to various neuromodulators.

The experiments revealed that certain signals could influence the overall activity of the cells. Quick-acting biogenic amines like octopamine and dopamine, for example, made IPCs release more insulin. These chemicals control energy use: dopamine is linked to motivating and reward, while octopamine shapes the response to stress and excitement, much like adrenaline in humans. As such, these signals likely help the body prepare for activity by ensuring enough resources are available. On the other hand, long-acting neuropeptides like leucokinin and myosuppressin reduced IPC activity and slowed insulin release. This may help conserve energy when food is scarce or during stress (Rosikon et al., 2023).

At the level of individual cells, the work by Held et al. confirmed that IPCs have heterogeneous roles in balancing metabolism, with some cells reacting strongly to certain neuromodulators while others remained unresponsive. This is similar to how human pancreatic beta cells behave, with various cells responding differently to various signals (Meyer et al., 2021). This diversity may help fine-tune insulin release, preventing sudden changes in blood sugar levels (Bisen et al., 2024; Held et al., 2025).

Taken together, these findings will enable further research into insulin regulation in fruit flies, which may hold the key to unlocking long-sought answers in how this process unfolds in humans. For instance, further studies could focus on exploring how the different signals controlling insulin release work together. Do some of them override others? And how do long-term changes, like obesity or diabetes, affect these signals? Answering these questions could lead to better treatments for metabolic diseases, such as diabetes.
